# Capturing 3D Water Flow in Rooted Soil by Ultra-fast Neutron Tomography

**DOI:** 10.1038/s41598-017-06046-w

**Published:** 2017-07-21

**Authors:** Christian Tötzke, Nikolay Kardjilov, Ingo Manke, Sascha E. Oswald

**Affiliations:** 1 Institute of Earth and Environmental Science, University of Potsdam, Potsdam, Germany; 2 Institute of Applied Materials, Helmholtz Centre for Materials and Energy, Berlin, Germany

## Abstract

Water infiltration in soil is not only affected by the inherent heterogeneities of soil, but even more by the interaction with plant roots and their water uptake. Neutron tomography is a unique non-invasive 3D tool to visualize plant root systems together with the soil water distribution *in situ*. So far, acquisition times in the range of hours have been the major limitation for imaging 3D water dynamics. Implementing an alternative acquisition procedure we boosted the speed of acquisition capturing an entire tomogram within 10 s. This allows, for the first time, tracking of a water front ascending in a rooted soil column upon infiltration of deuterated water time-resolved in 3D. Image quality and resolution could be sustained to a level allowing for capturing the root system in high detail. Good signal-to-noise ratio and contrast were the key to visualize dynamic changes in water content and to localize the root uptake. We demonstrated the ability of ultra-fast tomography to quantitatively image quick changes of water content in the rhizosphere and outlined the value of such imaging data for 3D water uptake modelling. The presented method paves the way for time-resolved studies of various 3D flow and transport phenomena in porous systems.

## Introduction

Non-invasive imaging techniques are the key for better understanding the root-soil interaction which is of great relevance for both plant and soil scientists. Neutrons are a unique probe for non-destructive investigation of root-soil systems. Unlike protons or X-rays, they interact only weakly with most materials, e.g. with most mineral soil particle, but strongly with hydrogen or, more precisely, hydrogen containing substances. For this reason, they can sensitively detect the presence of water and roots (which are mainly composed of water) in the soil matrix^1^. As neutrons also distinguish in interaction with different isotopes of hydrogen (essentially deuterium vs. hydrogen), water exchange processes inside porous soil matrix and the roots can be followed when deuterated water (D_2_O) is used as tracer substance^2^ while other contrast agents such as gadolinium still have to be established^3^. Neutron imaging has developed during the last decade into a powerful technique to study the architecture of root systems and the water distribution in the surrounding soil *in situ* ^4^. However, this is possible so far only by taking samples to a stationary neutron imaging facility, though there are some first approaches to develop also mobile neutron imaging devices^5^. The sample containers used have to be of material suited for a low level of neutron attenuation. Often aluminium is used, sometimes boron-free glass. Iron and other metals are possible, but only an inferior choice, whereas most common plastic materials are not a feasible choice because of being rich in hydrogen.

Due to the high temporal resolution (few seconds) 2D neutron radiography (NR) is capable to capture dynamic changes in the local water distribution of the sample. It has been successfully applied to visualize water transport phenomena in plants, e.g. root uptake^6, 7^, axial transport in the xylem^8^ and the formation of embolisms^9^. These studies provided valuable insights into the root uptake and axial water transport in plants. The analysis of water flows were, however, intrinsically restricted to the two-dimensional imaging approach. Extending the observation to three dimensions promises a great leap forward in understanding the water flow into the roots^4^. As the acquisition of a tomogram usually takes several hours insufficient time resolution has certainly been the greatest obstacle for visualizing three-dimensional flows by means of neutron tomography^10^, even more in rooted soil. A drastic acceleration of the acquisition process is, therefore, necessary to resolve the water dynamics of the rhizosphere in three dimensions. Dierick *et al*.^11^ have made a first attempt in this direction for infiltration in a rock sample, though with substantial compromises on spatial resolution. This approach has not been followed up any further, definitely not for plant-soil-systems, until a recent initiative by Zarebanadkouki *et al*.^12^. Instead the need for resolving roots and water content of surrounding soil has favoured the use of increasingly resolved images; with the downside that according tomography exhibits long acquisition times excluding the observation of dynamic processes such as water infiltration. We have consequently moved the existing approaches forward by modifying a number of experimental conditions towards high-speed tomography, enhanced by the available equipment, while not compromising too much on image resolution, signal-to-noise and contrast. A novel component introduced is the steady rotation of the sample accompanied by continuous acquisition of radiographic projections, which turned it into an ultra-fast tomography. The motion blur associated with the rotation during acquisition of the images has shown not to be a substantial limitation. This novel approach was tested in respect to quality of tomograms achieved and first results obtained for a quick wetting of a rooted soil and subsequent root water uptake, identified with help of deuterated water introduced.

## Results

### Progressive acceleration of acquisition speed

**Figure 1:**
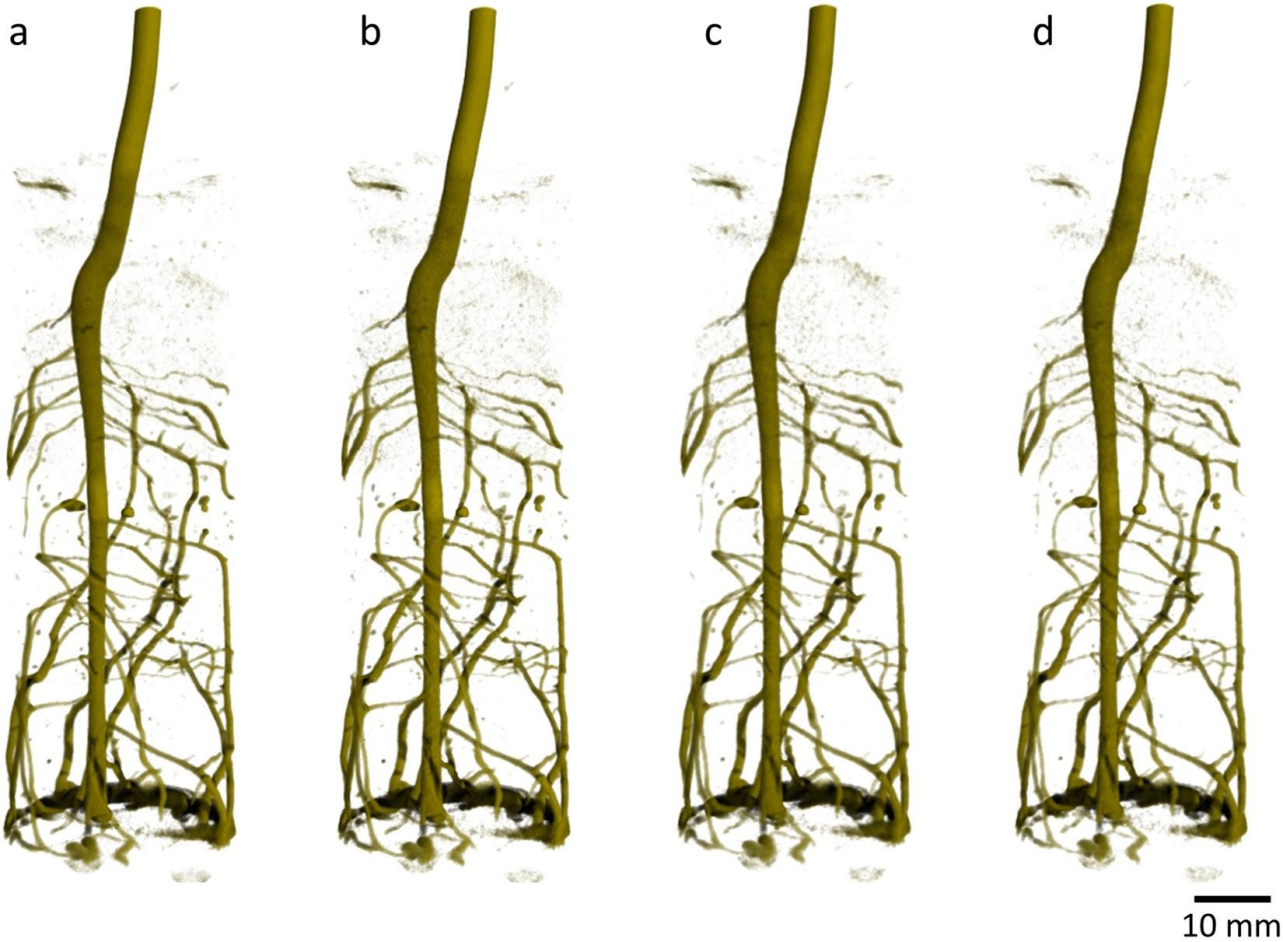
Lupine root system grown in sandy soil (total soil water content was 0.10 cm^3^/cm^3^) captured by ultra-fast neutron tomography at increasing speed of measurement. Total acquisition times were (**a**) 60 s, (**b**) 30 s, (**c**) 15 s (**d**) 10 s, and all 3D views are shown from the same perspective. Even at highest speed the major features of the root system are retained.

**Figure 2:**
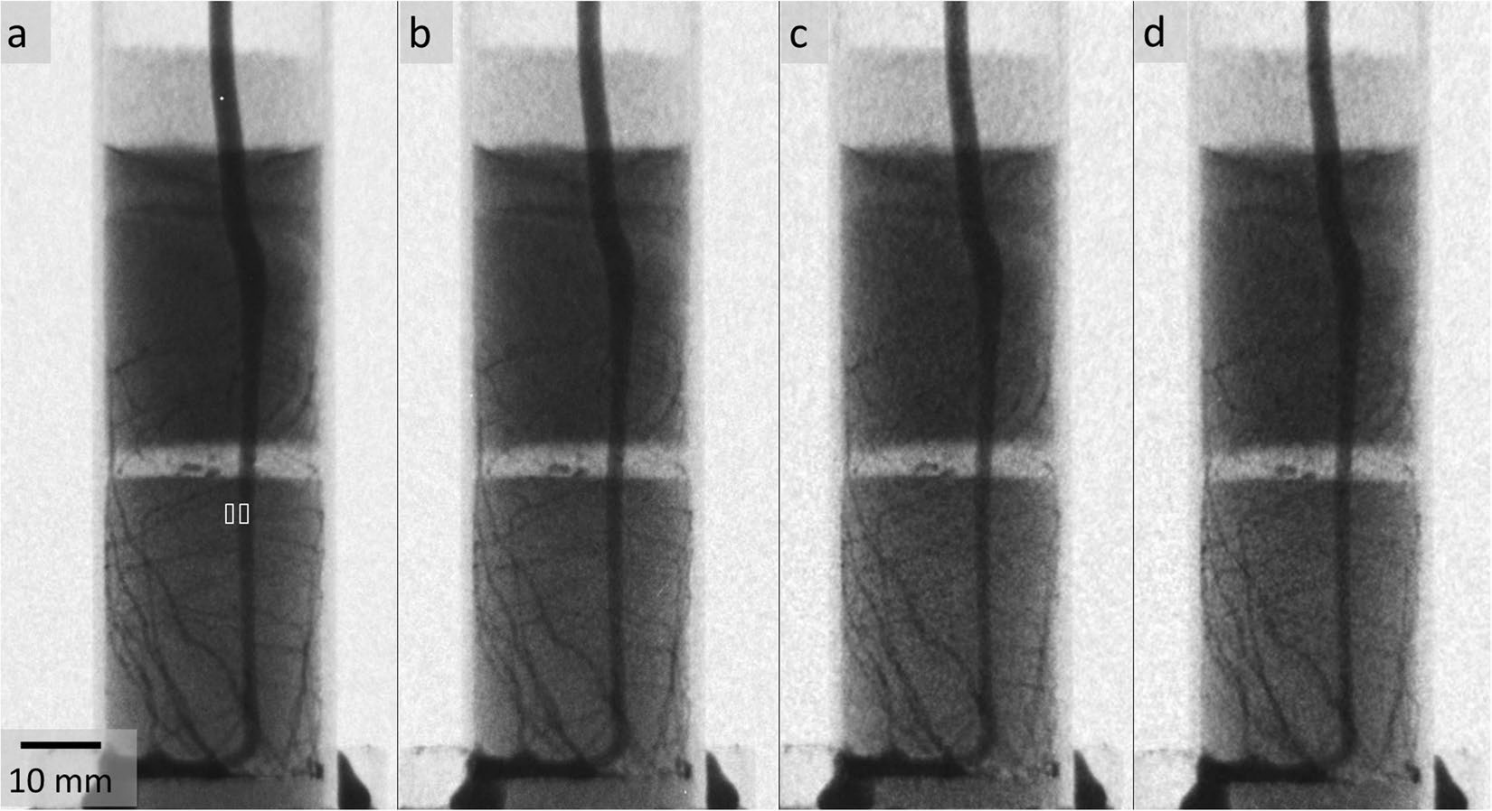
Analogous radiographic projections captured while sample was rotating in four different ultra-fast neutron tomographies constituting a tomographic test series. (**a**) 60 s – tomography: t_exp_ = 0.2 s; (**b**) 30 s – tomography: t_exp_ = 0.1 s; (**c**) 15 s – tomography: t_exp_ = 0.05 s; (**d**) 10 s – tomography: t_exp_ = 0.05 s. The white rectangles in a indicate regions used for contrast calculation.

In an initial test series the acquisition time per tomogram was progressively reduced from 60 s to 10 s and the quality of resulting images was evaluated. Figure 1 shows 3D renderings of the lupine root system as retrieved from the reconstructed sample volume. The images impressively demonstrate that the architecture of the root system can be captured in detail in less than one minute. Note, that already the slowest acquisition time (Fig. 1a; t_acquisition_ = 60 s) represents a speed up by a factor 100 with respect to standard neutron tomography. While further reducing the acquisition time by factor 6 (from left to right in Fig. 2) the major information about the root system architecture could be widely retained, i.e. shape and orientation of tap root and laterals roots are clearly visible.

### Image signal-to-noise and contrast of ultra-fast tomography

Our approach to speed up tomographic measurements is based on a modified acquisition procedure, which involves the continuous rotation of the sample as well as reduction of exposure time and number of radiographic projections. In the following we briefly discuss how this approach affects image quality in terms of spatial resolution and signal-to-noise ratio (SNR). An important general requirement for fast measurements is a high neutron flux available at the instrument, since the number of detected neutrons linearly decreases with reduction of exposure time. Accepting moderate increase in geometrical blur the enlargement of the pinhole aperture is a useful measure to increase the neutron flux at the detector. Using a 3 cm aperture the physical spatial resolution of the detector system (exposure time t_exp_ = 0.2 s, L/D = 167, no binning) was about 150 µm (see test pattern depicted in Fig. 1). Applying 2 × 2 and 3 × 3 binning the resolution reduced to about 200 and 280 µm mode while the effective signal intensity was enhanced by factor 4 and 9, respectively.

Figure 2 displays one radiographic projection for each tomogram measured in the initial test series. Noise and resolution are on a reasonable level to resolve the major details of the root architecture even at the highest speed of acquisition. From the visual inspection of Fig. 2a–d a moderate increase of noise and slight deterioration in sharpness of details is noticed with increasing speed of acquisition. The signal-to-noise ratio (SNR = (signal intensity − background intensity)/standard deviation of signal) was exemplarily calculated for radiographic projections and their corresponding flatfield images. The signal intensity was determined for a 20 × 50 mm^2^ soil-filled region in the central part of the sample and the standard deviation for a 7 × 7 mm^2^ region at the centre of the flatfield. The SNR of flatfields ranged from 17.9 to 20.8 while the SNR of projections ranged from 4.8 to 7.0 (see Table 1). Note that the soil water content - being the dominating factor for neutron attenuation by the sample - strongly affects signal intensity and hence the SNR of the projection. For comparison, we also calculated SNR for a plant sample with identical dimensions and similar soil water content (θ = 0.09 cm^3^cm^−3^) which was measured by conventional tomography in previous experiments at spatial resolution of 40 µm/pixel (not shown here). SNR were higher (21.4 for flatfield and 10.7 for projection) but still in a range comparable to those of the ultra-fast acquisition. The conventional measurement had a better physical resolution of about 100 µm/pixel, however the temporal resolution (t_exp_ = 10 s/projection and 100 min/tomogram) was more than 100-fold lower. Thus, the gain in time is substantially higher than is the loss in space.

**Table 1:** Overview on signal to noise ratios and contrast between roots and soil measured for the initial test series.

t_exp_ [s/projection]	SNR flatfield	SNR projection	Contrast root-soil (radiograph), %	Contrast root-soil (tomographic slice), %
0.2	28.0	7.0	18	40
0.1	20.8	5.6	17	38
0.05	17.9	4.9	16	41
0.05	17.9	4.8	14	43

A specific advantage for imaging samples with rooted soil is the strong contrast between roots and soil. However, in 2D radiographs this contrast is diluted because each pixel represents already a mixed signal of root and soil water^13^. This makes 3D tomography very attractive, because the latter has the potential to maintain the original contrast and extract the water content changes at the important interface between roots and soil much better. We exemplary calculated the contrast between tap root and soil for the radiographic projection and the according tomographic slice after 3D reconstruction, respectively,

$$Contrast=\frac{{I}_{soil}-{I}_{root}}{{I}_{soil}+{I}_{root}}\cdot 100\, \% $$

with *I* being the signal intensity (regions indicated in Fig. 2a). The contrast ranged between 14% and 18% in the radiographs and 38% and 43% in the tomographic slices (not shown here). These high contrast values facilitate capturing the root system in detail also at high speed.

### Blurring of images by continuous sample rotation

**Figure 3:**
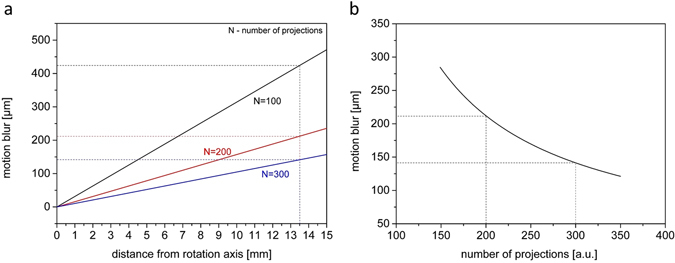
Continuous sample rotation results in motion blur of radiographic projections. Motion blur increases linearly with distance from rotation axis (**a**) and is inversely proportional to the number of projections (**b**).

A specific feature of the presented ultra-fast neutron measurements is the continuous rotation of the sample during tomographic acquisition. This inevitably adds some motion blur to the radiographic images. The degree of motion blur depends on the object speed in relation to the exposure time and the spatial resolution of the image. During acquisition, the number of radiographic projections taken over a specific angular interval determines this relative speed. Considering a rotational movement, motion blur within the sample increases linearly with distance from the rotation axis. In Fig. 3a this fundamental relationship is plotted for different numbers of projections. In addition, Fig. 3b displays the maximum motion blur occurring at the outer edge of the considered sample (Ø27 mm) for increasing number of projections. The motion blur occurring at this distal edge of the sample is 140 µm for 300 projections and 212 µm for 200 projections. As these values are below the spatial resolution they do not substantially deteriorate the image quality and can be tolerated.

The contrast provided from the tomographic slices is related to the difference of the attenuation coefficients in these slices. The small deviations between the values in Table 1, last column, show that the reconstruction of the attenuation coefficients is reliable even if the SNR in the angular projections is reduced (see 3^rd^ column in Table 1).

### Tracking dynamic water movement in a rooted soil sample

**Figure 4:**
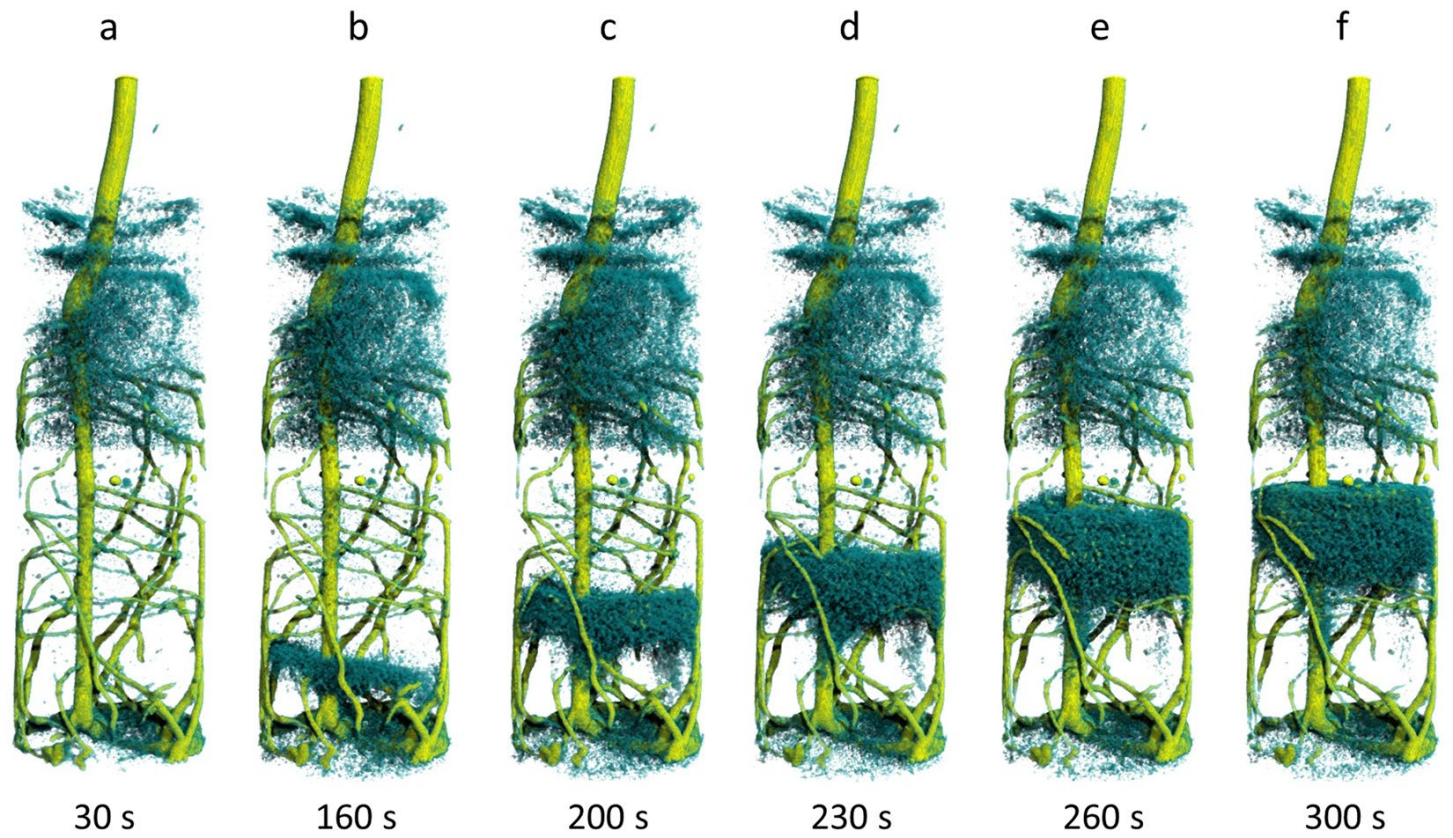
Time-resolved neutron tomography of the lupine root system after the injection of 4 ml deuterated water (D_2_O) through the bottom. The time series (30 s ≤ t ≤ 300 s) shows the ascending front of water (H_2_O) displaced by deuterated water moving upwards. The time resolution for each tomogram is just 10 s. A video of this sequence is provided in the supplementary information.

In the second part of the experiment, we tested the capability of the acquisition procedure to track dynamic water flows through the porous soil column. The 4 ml of deuterated water applied at the bottom quickly entered the sample as it was lifted upwards by the capillarity of pores, first through the porous bottom plate and then into the soil. In Fig. 4 we visualized the water front rising upwards or, more precisely, the displacement of light water being pushed upwards by deuterated water.

Water content isosurfaces were retrieved using the volume renderer ‘SquatterHQ’ of VGStudio MAX. A video of this sequence is provided in the supplementary information (see Video 1) of this article. During adjustment of renderer settings the histogram was divided by thresholds in three intervals in order to visually distinguish between roots, water and soil. Voxels with the highest attenuation (corresponding to a local water content θ > 0.5) were rendered in yellow representing the root tissue, those with medium local attenuation values (corresponding to a soil water range of about 0.1 < θ < 0.5, i.e. less strongly bound and better plant available water) in turquois representing soil water. Voxels with lower attenuation were rendered transparent, because they represent wall material or drier pores with fewer and more strongly adhering water.

Figure 4a displays the initial state of the rooted soil column, which is hydraulically separated by a layer of coarse sand at medium height. As indicated by the turquois cloud (representing the 3D soil water distribution above the threshold) the upper soil compartment is significantly wetter than the bottom compartment. Since the soil in the lower compartment is relatively dry (water content below 0.1 cm^3^/cm^3^) it is rendered transparent. This low soil water content implies that the soil has dried to a level where the remaining water is almost not available for plant uptake by lupine roots anymore and water uptake will cease^14^.

Upon injection of deuterated water (Fig. 4b), the water still present in soil pores is successively displaced. Though deuterated water itself is not visible due to its low attenuation coefficient (µ_H2O_ = 5.4 cm^−1^ vs. µ_D2O_ = 0.1 cm^−1^) its movement can be detected by the accumulation of soil water above the ascending deuterated water front, being tracked as turquois, cloudy structure in Fig. 4. With further progress the front of deuterated water displaced soil water not only moves upwards, but its total amount increases correspondingly, as visible in form of the expansion of the region with higher water content shown in turquois. The relatively large amount of soil water displaced has to be interpreted as almost complete wetting of soil pores by D_2_O with according replacement of the water that was present before. This has already been observed in 2D experiments^15^ but in the 3D tomography the high degree of water replaced becomes clearer and local deviations by roots or walls can be identified. After 5 minutes, the upper front of displaced water has reached the top of the lower soil compartment where it is sharply stopped by the capillary barrier layer (Fig. 4f).

**Figure 5:**
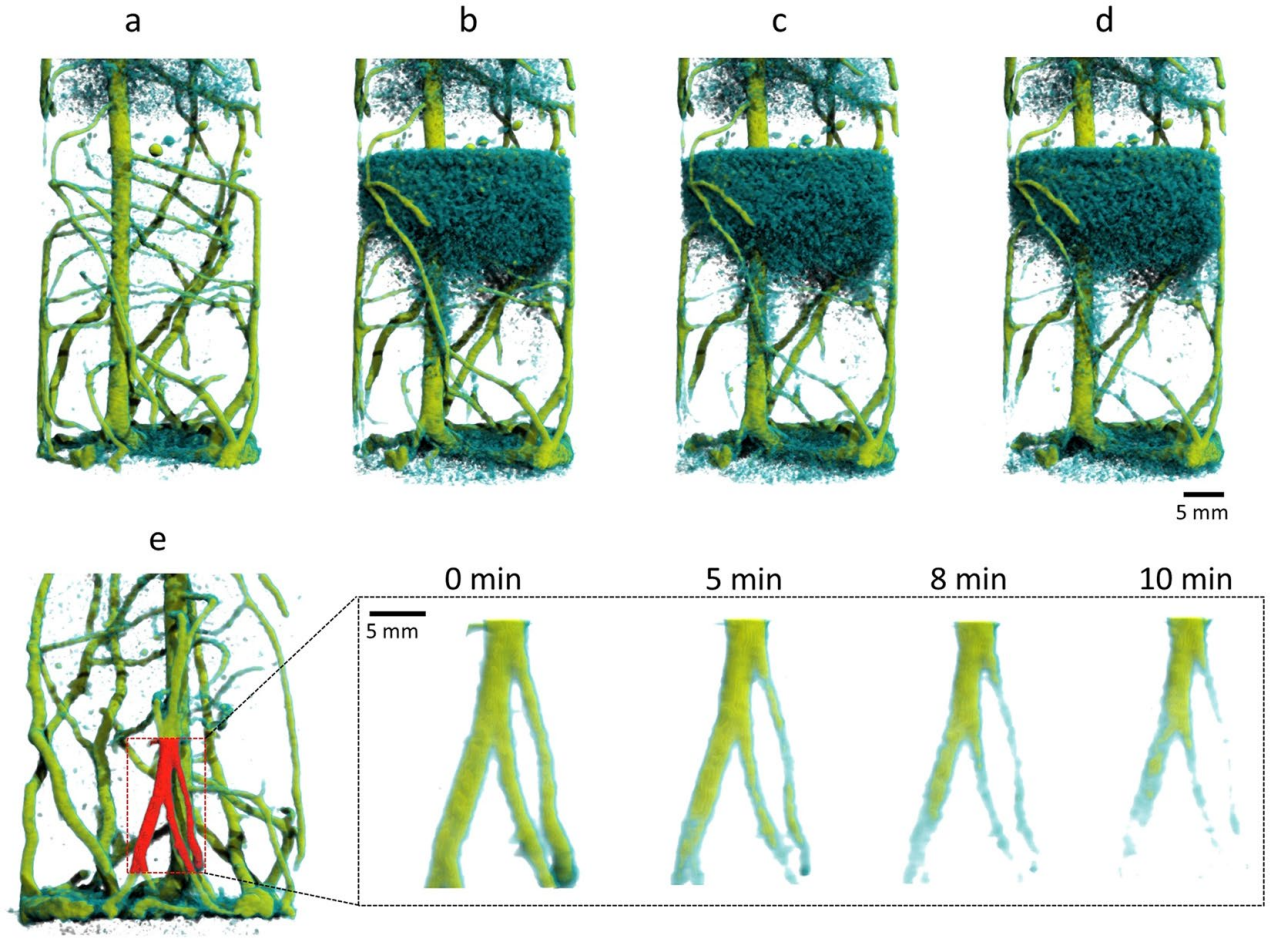
Time series of neutron tomograms showing the lower soil compartment of the plant container at (**a**) 0 min; (**b**) 5 min; (**c**) 8 min (**d**) 10 min after feeding D_2_O through the bottom plate. In the bottom row three lateral root segments are selected (area indicated in **e**) and shown as a close-up time development. The gradual disappearance of the lower part of the lateral roots indicates the uptake of deuterated water by these root segments.

The stable shape and amount of the water cloud in the following period 5 to 10 minutes after injection started (Fig. 5b–d) indicates a relatively stagnant 3D distribution of the soil water replaced. This confirms that the local dispersion in the porous soil matrix is small and mixing of the deuterated with non-deuterated water does only proceed slowly. This also allows observing a second phenomenon during this period, i.e. the uptake of deuterated water by lateral roots.

### Identifying root water uptake

When entering roots the deuterated water successively displaces H_2_O in the roots causing a decrease of local neutron attenuation of root tissue. Figure 5 displays a time series of the lower soil compartment over a course of 10 minutes using identical rendering settings as in Fig. 4. Taking reference to the initial state depicted in Fig. 5e lateral roots start to gradually disappear from the bottom of the container 5 minutes after the start of injection of deuterated water (Fig. 5e, close-up). This development continues indicating active water uptake especially in the apical region of the lateral roots. On longer time scales also the movement of deuterated water inside the roots could be tracked, becoming distinguishable especially after passing the capillary barrier layer, as was done in two-dimensional radiography recently^15^.

## Discussion

Pushing the limits of neutron tomography towards much faster acquisition speeds as main objective turned out to be remarkably successful, with very moderate compromises on signal-to-noise, spatial resolution and contrast. Taking advantage of the high and constant neutron flux available at the upgraded CONRAD II imaging instrument we sped up the acquisition of neutron tomograms from hours to tens of seconds. Progressive reduction of acquisition time from 60 s to 10 s only resulted in a slight deterioration of image quality and the modified acquisition procedure did not introduce additional imaging artefacts. The tremendous gain in measurement speed clears the way for time-resolved 3D measurements of dynamic changes in water distribution. We could demonstrate this for the first time by a tracking of a water front ascending in a column of rooted soil upon infiltration of deuterated water from the bottom. The image quality was maintained even at maximum acquisition speed such that the root architecture could be resolved in high detail. Furthermore, the root uptake of D_2_O was proven in particular for apical root regions. Overcoming the problem of long acquisition time, ultra-fast neutron tomography opens up the possibility to image the 3D water dynamics of the rhizosphere. Thus, the water distribution in soil can be mapped with good reproducibility (Fig. 5, top part of sample) and be distinguished from roots and dry pore space, which is much more challenging for alternative 3D approaches^16, 17^. Compared to conventional neutron tomography another advantage is the much shorter exposure time to the neutron beam. For 2D neutron radiography basic estimates show that radiation side-effects are probably not an issue^18^, while the conventional tomography is already at an exposure level closer to possible damages of plants due to the much longer exposure times. Whereas the ultra-fast neutron tomography introduced has only an exposure in order of a 2D neutron radiography and could be even repeated several times, though not continuously, while still minimizing radiation damages.

So far the observation of root uptake via D_2_O tracing was only possible by 2D neutron radiography with the intrinsic drawback of losing the depth information in beam direction. Using ultra-fast neutron tomography we are now able to detect and exactly localize the D_2_O uptake by individual root segments time-resolved in three dimensions. Further studies could also track the transport of the D_2_O towards tap root and even into the stem. Altogether, capturing root system architecture and resolving dynamic root uptake and transport in a three-dimensional way, ultra-fast tomography offers a great chance to enhance the predictive power of up-to-date water flow and transport models. Sophisticated 3D approaches modelling root-soil interactions calculate water and nutrient transport under consideration of the specific architecture of the root system^19–21^. The information necessary about architecture and transport dynamics can now be retrieved from such tomographic data and thus model parameterization and validation can now be based on the actual root system that drives the uptake^10^. Both, time-lapsed experimental 3D tomography data as well as associated numerical simulation of dynamic changes in 3D root-soil systems will create huge data sets that create a need for specific methods in processing, visualization and interpretation of these big data.

The present study demonstrated the feasibility of ultra-fast neutron tomography to visualize 3D flows in porous media, which suggests further applications in the field of soil, material and geosciences^22^. It could be used to study transport phenomena in sand, soil or rocks, e.g. preferential flow in structured soil^23^, fingered flow in hydrophobic sand^24^, non-wetting phase entrapments by snap-off^25^ or transport through fractured rocks^24, 26^, now with unprecedented temporal resolution. The method can be assisted by the application of D_2_O pulses providing contrast in saturated pore matrixes, which is necessary to differentiate between stationary and moving water.

Finally, enhancement of the neutron beam intensity holds great further potential for ultra-fast tomography. When applied at even more powerful neutron facilities compared to BER II, such as the continuous neutron source at ILL in Grenoble, France (6 times higher power) or the future ESS pulsed neutron source in Lund, Sweden (6 times higher intensity), the resolution capability of the method can be further improved. Greater neutron flux could be either exploited for acceleration of the acquisition process or for refining the spatial resolution in single images. Overall, scientists now have the choice between investing a considerable time for a tomography with ultimate quality and resolution limited to a stagnant water distribution, or obtaining 3D data of at least acceptable quality but tracking quick changes of water redistribution in soil and associated root water uptake. Notwithstanding, a combination of the two types of 3D imaging could be the most fruitful approach, e.g. by first retrieving the 3D root system in highest quality before tracking water dynamics with the novel ultra-fast tomography.

## Methods

We performed non-invasive imaging experiments on young, potted lupine plants. Plants were cultivated at the University of Potsdam whereas the imaging experiments were carried out nearby at the neutron source BER II of Helmholtz Centre Berlin using the CONRAD II cold neutron imaging instrument^27^.

### Plant growing

Individual white lupine plants (*lupinus albus*) were grown in quartz glass columns of 27 mm diameter and 100 mm height filled with sandy soil collected at the Chicken Creek catchment near Cottbus, Germany (cf.^14, 28^). At medium height, the soil column was compartmented by a 1 cm thick horizontal layer of coarse sand, which blocked capillary water transport between the compartments without hindering roots to grow across (see ref. 14). Seeds were placed on wet paper towels in the dark for 24 hours to start germination, and then planted at 1 cm soil depth. After sprouting an additional 1 cm thick layer of gravel was added to the top in order to minimize evaporation from the soil surface. Plants were grown in a plant growth chamber applying a photoperiod of 14 h per day with light intensity of 400 µmol/(m^2^s). The day and night temperatures were 24 °C and 19 °C, respectively, while the relative humidity was kept at 60%. Plants were irrigated every second day from top and bottom to a water content of 0.25 cm^3^/cm^3^. The imaging experiments started 11 days after planting. During the tomographic acquisition plants were illuminated from above by a plant growing lamp (light intensity also 400 µmol/(m^2^s)) to sustain transpiration.

### Imaging experiments

The CONRAD II imaging facility is provided with a high and very stable neutron flux by the cold neutron source of the BER II research reactor. For this reason, it offers best technical conditions for performing fast neutron tomography. We modified the established experimental procedures in several ways to achieve as short acquisition times for a tomogram as possible. 1) The neutron flux was enhanced by adjusting the pinhole to allow for a bright illumination by neutrons, though losing some resolution. 2) All projections were stored in the built-in memory of the camera, which was read out only after complete acquisition. 3) The radiographic projections were captured on the fly during the sample is rotating, avoiding the time-consuming stepwise rotation with iterated acquisition of individual projections, which has been the most limiting factor for achieving tomograms with high speed^11^. Overall, a fine-tuned balance was kept to sustain the quality of tomograms sufficient for investigation the root-soil interface, though reducing the time spent for acquisition tremendously.

In detail, a detector system consisting of a 200 µm thick ^6^LiZnS:Ag scintillator screen and sCMOS camera (Andor ‘Neo’) in combination with a bright Nikon photo lens (focus 50 mm, aperture 1.2) was employed at the middle measure position of the instrument where beam conditions are suitable for high speed measurements^27, 29^. The beam path length from the collimator to the sample position was 5 m, the pinhole aperture 3 cm resulting in an L/D ratio of 167. The physical spatial resolution of the employed detector system was about 150 µm (exposure time 0.2 s, no binning, see test pattern depicted in Figure S1 of the supplementary information). Pixel binning (2 × 2 or 3 × 3) was applied to enhance signal-to-noise ratio resulting in effective pixel sizes of 110 and 165 µm, respectively.

For the image acquisition, we used a LabVIEW-based software system to control the interaction of the individual components of the imaging instruments. The plant sample was placed and secured on an aluminium dish serving as water reservoir during irrigation. This dish was mounted on a manipulation stage in front of the scintillator. In contrast to conventional tomographic acquisition in which the sample is stepwise revolved over a range of 180° or 360° we rotated the sample at constant speed while images were taken continuously. The speed of rotation was adjusted such that 300 or 200 radiographic projections were taken over a range of 180° during the desired total acquisition time of the tomogram of the sample. In an initial test series we stepwise reduced the acquisition times from 60 s to 10 s per tomogram. Exposure time, binning factor and number of projections used in this series are summarized in Table 2. Afterwards, the plant sample was watered from below with a predefined amount of water (4 ml) fed into the aluminium dish holding the plant container by a syringe pump (Fresenius Pilot C) at a rate of 100 ml/h. We applied deuterated water (D_2_O) instead of H_2_O in order to trace and localize the uptake of D_2_O by individual root segments. The ascending water front and subsequent root uptake were measured as time-lapsed series in three dimensions. After acquisition, self-programmed IDL-routines were used to arrange the image files into the required folder structure needed by the next processing step. Tomograms were reconstructed and rendered in 3D using the software tools Octopus (Inside Matters, Gent/Belgium) and VGstudio Max (Volume Graphics, Heidelberg/Germany).

**Table 2:** Imaging parameter during initial test series.

Exposure time [s/projection]	Binning factor	Effective pixel size [µm/pixel]	Number of projections	Acquisition time [s/tomogram]
0.20	2 × 2	110	300	60
0.10	2 × 2	110	300	30
0.05	3 × 3	165	300	15
0.05	3 × 3	165	200	10

## Electronic supplementary material

Video 1

Supplementary Information

## References

[1] Moradi AB (2009). Root responses to soil Ni heterogeneity in a hyperaccumulator and a non-accumulator species. Environ. Pollut..

[2] Kardjilov N, Manke I, Hilger A, Strobl M, Banhart J (2011). Neutron imaging in materials science. Mater. Today.

[3] Oswald SE (2015). Combining Neutron and Magnetic Resonance Imaging to Study the Interaction of Plant Roots and Soil. Phys. Procedia.

[4] Moradi AB (2011). Three-dimensional visualization and quantification of water content in the rhizosphere. New Phytol..

[5] Buecherl, T., Kardjilov, N., Gostomski, C. L. v., Calzada, E. & El Ghobary, A. M. A mobile neutron source based on the SbBe reaction. *Appl. Radiat. Isot*. **61**, 659-662, doi:101016/japradiso200403094 (2004). 10.1016/j.apradiso.2004.03.094 15246414

[6] Zarebanadkouki, M. *et al*. Quantification and Modeling of Local Root Water Uptake Using Neutron Radiography and Deuterated Water. *Vadose Zone J*. **11**, doi:10.2136/vzj2011.0196 (2012).

[7] Rudolph-Mohr, N., Tötzke, C., Kardjilov, N. & Oswald, S. E. Mapping water, oxygen, and pH dynamics in the rhizosphere of young maize roots. *J. Plant Nutr. Soil Sci*., **180**, 336–346, doi:10.1002/jpln.201600120 (2017).

[8] Matsushima U (2009). Estimation of water flow velocity in small plants using cold neutron imaging with D2O tracer. Nucl. Instrum. Methods Phys. Res., Sect. A.

[9] Tötzke C (2013). Visualization of embolism formation in the xylem of liana stems using neutron radiography. Ann. Bot..

[10] Roose T (2016). Challenges in imaging and predictive modeling of rhizosphere processes. Plant Soil.

[11] Dierick M (2005). High-speed neutron tomography of dynamic processes. Nucl. Instrum. Methods Phys. Res., Sect. A.

[12] Zarebanadkouki M (2015). On-the-fly Neutron Tomography of Water Transport into Lupine Roots. Phys. Procedia.

[13] Oswald S.E. (2008). Quantitative Imaging of Infiltration, Root Growth, and Root Water Uptake via Neutron Radiography. Vadose Zone Journal.

[14] Dara A, Moradi BA, Vontobel P, Oswald SE (2015). Mapping compensating root water uptake in heterogeneous soil conditions via neutron radiography. Plant Soil.

[15] Zarebanadkouki M, Kim YX, Carminati A (2013). Where do roots take up water? Neutron radiography of water flow into the roots of transpiring plants growing in soil. New Phytol..

[16] Downie HF (2015). Challenges and opportunities for quantifying roots and rhizosphere interactions through imaging and image analysis. Plant, Cell Environ.

[17] Wildenschild D, Vaz CMP, Rivers ML, Rikard D, Christensen BSB (2002). Using X-ray computed tomography in hydrology: systems, resolutions, and limitations. J. Hyd.

[18] Menon M (2007). Visualization of root growth in heterogeneously contaminated soil using neutron radiography. Eur. J. Soil Sci..

[19] Pagès L (2011). Links between root developmental traits and foraging performance. Plant, Cell Environ.

[20] Dunbabin VM (2013). Modelling root–soil interactions using three–dimensional models of root growth, architecture and function. Plant Soil.

[21] Huber K (2014). Modelling the impact of heterogeneous rootzone water distribution on the regulation of transpiration by hormone transport and/or hydraulic pressures. Plant Soil.

[22] Perfect E (2014). Neutron imaging of hydrogen-rich fluids in geomaterials and engineered porous media: A review. Earth-Sci. Rev..

[23] Gerke HH (2006). Preferential flow descriptions for structured soils. J. Plant Nutr. Soil Sci..

[24] Ritsema CJ, Dekker LW (1994). How water moves in a water repellent sandy soil: 2. Dynamics of fingered flow. Water Resour. Res.

[25] Berg S (2013). Real-time 3D imaging of Haines jumps in porous media flow. Proc. Natl. Acad. Sci. USA.

[26] Neuman SPT (2005). prospects and challenges in quantifying flow and transport through fractured rocks. Hyd. J.

[27] Helmholtz-Zentrum Berlin für Materialien und Energie. CONRAD-2: Cold Neutron Tomography and Radiography at BER II (V7). *J.L.S.R.F*. **2**, A98, doi:10.17815/jlsrf-2-108 (2016).

[28] Moradi, A. B. *et al*. Is the Rhizosphere Temporarily Water Repellent? *Vadose Zone J*. **11**, doi:10.2136/vzj2011.0120 (2012).

[29] Kardjilov N, Hilger A, Manke I, Woracek R, Banhart J (2016). CONRAD-2: the new neutron imaging instrument at the Helmholtz-Zentrum Berlin. J. Appl. Crystallogr..

