# Supplementary material for: Capturing 3D Water Flow in Rooted Soil by Ultra-fast Neutron Tomography
Source: Sci Rep. 2017 Jul 21;7:6192. doi: 10.1038/s41598-017-06046-w (PMC5522441; doi:10.1038/s41598-017-06046-w)
Supplement: Supplementary file 2 — Supplementary Information [file 41598_2017_6046_MOESM2_ESM.pdf]

Supplementary information to

"Capturing 3D Water Flow in Rooted Soil by Ultra-fast Neutron Tomography"

Christian Tötzke[1,*], Nikolay Kardjilov[2], Ingo Manke[2], Sascha E. Oswald[1]

[1]Institute of Earth and Environmental Science. University of Potsdam, Potsdam, Germany
[2]Institute of Applied Materials. Helmholtz Centre for Materials and Energy, Berlin, Germany

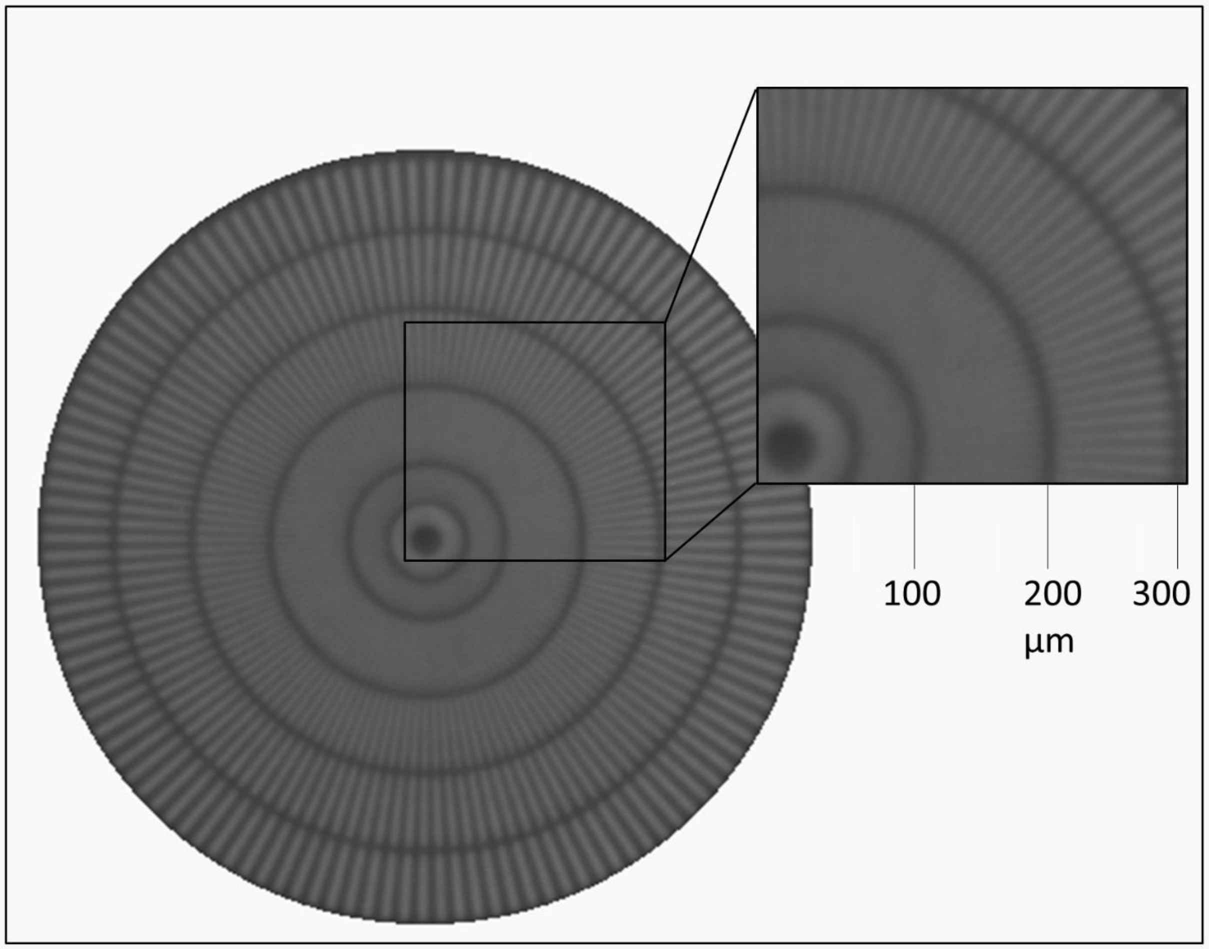

Figure S1: Physical spatial resolution of the applied detector system measured by a Siemens star.
